# Photobiomodulation of extracellular matrix enzymes in human nucleus pulposus cells as a potential treatment for intervertebral disk degeneration

**DOI:** 10.1038/s41598-018-30185-3

**Published:** 2018-08-03

**Authors:** Min Ho Hwang, Hyeong Guk Son, Jae Won Lee, Chang Min Yoo, Jae Hee Shin, Hyo Geun Nam, Hyun Jung Lim, Seung Min Baek, Jeong Hun Park, Joo Han Kim, Hyuk Choi

**Affiliations:** 1 Department of Medical Sciences, Graduate School of Medicine, Korea University, Seoul, Korea; 2 Department of Neurosurgery, Guro Hospital, College of Medicine, Korea University, Seoul, Korea

## Abstract

Intervertebral disc (IVD) degeneration is associated with imbalances between catabolic and anabolic responses, regulated by extracellular matrix (ECM)-modifying enzymes such as matrix metalloproteinases (MMPs) and their endogenous tissue inhibitors of metalloproteinases (TIMPs). Potential contributing factors, such as interleukin (IL)-1β and tumor necrosis factor (TNF)-α, derived from infiltrated, activated macrophages within IVD tissues, can trigger abnormal production of ECM-modifying enzymes and progression of IVD degeneration. Novel therapies for regulating ECM-modifying enzymes can prevent or ameliorate IVD degeneration. Photobiomodulation (PBM), known to regulate wound repair, exhibits regenerative potential by modulating biological molecules. This study examined the effects of PBM, administered at various wavelengths (630, 525, and 465 nm) and energy densities (16, 32, and 64 J/cm^2^), on the production of ECM-modifying enzymes in replicated degenerative IVD. Our results showed that PBM selectively inhibited the production of ECM-modifying enzymes in a dose- and wavelength-dependent manner, suggesting that it could be a novel tool for treating symptomatic IVD degeneration.

## Introduction

Chronic low back pain (LBP) affects approximately 632 million people during their lifetime, resulting in a large socio-economic burden, with annual costs exceeding $100 billion in the United States alone^1–3^. Although the factors causing LBP in the lumbar spine, including mechanical trauma, genetics, and infection, are multifactorial, a significant proportion of LBP cases are strongly linked to intervertebral disc (IVD) degeneration^4,5^. Current strategies are restricted to surgical intervention or conservative therapies. These approaches are focused on alleviating the symptoms by inducing temporary analgesia, rather than on exploring the mechanisms underlying the etiology of painful IVD^6^. Neurovascular ingrowth is consistently identified in degenerative IVD by histologic analysis, and is a possible cause of LBP. Intact and abundant matrix components, such as collagen and aggrecan, inhibit these phenomena in the healthy state^2,7,8^. To derive novel treatment modalities, it is essential to understand the process of degeneration responsible for the degradation of matrix components.

The IVD is part of an anatomic unit that includes the centrally located nucleus pulposus (NP) and peripherally located annulus fibrosus (AF). It is normally avascular and aneural in a healthy state. The IVD plays a major role in human physiological movement by providing flexibility to the spine and resistance to spinal compression. This movement enables the maintenance of matrix turnover and nutrient supply into the IVD^9–11^. However, immoderate biomechanical loading leads to structural disruption, resulting in an inflammatory response and degenerative condition. The degradation of matrix components, stemming from these changes implicated in degenerative IVD, provides a permissive microenvironment that favors neuronal ingrowth and neovascularization^12–15^.

The structural deficits occurring during degeneration, including the formation of tears and clefts in IVD tissues, permit the infiltration and activation of immune cells, including macrophages, neutrophils, and monocytes. The infiltrating immune cells continue to release proinflammatory cytokines, such as interleukin (IL)-1β and tumor necrosis factor (TNF)-α, which can trigger a range of pathogenic responses in IVD cells^16,17^. IL-1β and TNF-α, which are secreted by the immune cells, induce upregulation of genes that encode extracellular matrix (ECM)-modifying enzymes^18,19^. Matrix degradation is regulated by extracellular matrix-modifying enzymes such as matrix metalloproteinases (MMPs) and tissue inhibitors of metalloproteinases (TIMPs)^20,21^. Degenerated IVD tissue shows a marked increase in the production of MMPs in the NP region. The presence of MMPs poses etiological questions on whether modulating these ECM-modifying enzymes can ameliorate or prevent symptomatic IVD degeneration^22,23^. Furthermore, a previous study reported that deep nerve ingrowth into the inner NP, resulting from degradation of ECM components as a first line of defense against nerve ingrowth, is associated with LBP via the expression of nociceptive neurotransmitters, such as substance P, in IVD tissues. That study demonstrated that nerve ingrowth in the NP region is more important than that in the AF region in LBP or in pathogenesis of IVD degeneration^24^. Thus, the regulation of ECM-modifying enzymes in NP cells may be a novel therapeutic target for relieving LBP.

Light-activated therapeutic applications exploit the versatility of optics in modulating proteins, photoactive molecules, and cells. PBM, refers to the use of photons at a non-thermal irradiance to regulate biological activity. Moreover, PBM is efficacious for enabling tissue repair, exerts anti-inflammatory effects, and promotes wound healing in various cells^25–35^. During this process, a molecule excited by PBM can interact with a neighboring molecule to enhance photobiological effects, including regulation of a target gene, destruction of enzymes in a catabolic response, and secretion of a specific protein by target cells. PBM exerts anti-inflammatory effects by regulating reactive oxygen species (ROS) and decreasing the expression of IL-1β mRNA^36,37^. Furthermore, PBM is involved in modulating the effects of MMP activity and collagen synthesis^38^, which are major ECM components in the IVD. Although evidence with respect to positive effects of PBM is accumulating, the molecular mechanisms of PBM are not well understood. Moreover, the regulatory effects of PBM on the production of ECM-modifying enzymes, which are induced by interactions between macrophages and human NP cells, have not been studied. In addition, the effects of PBM depend on the use of optimal parameters such as wavelengths and energy densities.

Previous studies from our laboratory have shown that PBM selectively modulates the ECM-modifying enzymes and inflammatory mediators in inflamed human AF cells, which are the dominant interactions or responses in the early stages of IVD degeneration^26,27^. However, the effects of PBM on human NP cells have not yet been studied. In addition, due to its histological location within IVD tissues, human NP cells may have a different role in the etiology of IVD degeneration, especially in the late stages. Hence, we questioned whether the expressed matrix enzymes from the inflammation model of nucleus pulposus may be regulated by PBM and explored the differences with that in annulus fibrosus model.

In this study, we hypothesized that macrophages can induce degenerative conditions by secreting ECM-modifying enzymes and leading to regulation of genes encoding catabolic enzymes in human NP cells. To explore optimal parameters and verify the effect of PBM on human NP cells, we studied a spectrum range of 465, 525, and 630 nm, and doses of 16, 32, and 64 J/cm^2^.

## Materials and Methods

### Isolation and culture of human NP cells

Human NP cells were obtained from IVD tissues removed from consenting patients during surgical procedures. The tissues were obtained according to the regulations and all experimental protocols were approved by the institutional review board of Korea University Hospital (KUGH17208-001). Written informed consent was obtained from the patients. All methods were carried out in accordance with relevant guidelines and regulations. IVD tissue specimens were placed into sterilized Ham’s F-12 medium (Gibco-BRL) supplemented with 5% fetal bovine serum (FBS; Gibco-BRL) and 1% penicillin/streptomycin (P/S; Gibco-BRL). After washing the tissues, the definitive NP regions were dissected and digested for 60 min in F-12 medium containing 1% P/S, 5% FBS, and 0.2% Pronase (Calbiochem, La Jolla, CA, USA), followed by incubation for 24 h in a medium containing 0.025% collagenase. Cells were filtered using a sterile nylon mesh (70-μm pore size) to remove tissue debris and isolate human NP cells. The isolated NP cells were cultured in 75-cm^2^ cell culture flasks (VWR Scientific Products, Bridgeport, NJ, USA) in a humidified atmosphere with 5% CO2 at 37 °C.

### Differentiation of human monocytic leukemia THP-1 cells into activated macrophage-like cells and generation of macrophage-conditioned medium (MCM)

The human monocytic leukemia THP-1 cell line (ATCC TIB202; ATCC, Manassas, VA, USA) was seeded into 75-cm^2^ cell culture flasks containing Roswell Park Memorial Institute (RPMI) 1640 medium supplemented with 160 nM phorbol myristate acetate (PMA), 1% FBS, and 1% P/S. After 48 hours, activated macrophage-like THP-1 cells continued to secrete proinflammatory cytokines, such as IL-1β and TNF-α, which can trigger degenerative conditions. The cells were washed with phosphate buffered saline (PBS; Gibco-BRL), and cultured in Dulbecco’s Modified Eagle Medium: Nutrient Mixture F-12 (DMEM/F12) containing 1% FBS and 1% P/S for 48 hours. To justify the effectiveness of NF-κB inhibitor, cells were pre-treated with BAY11-7082 (Sigma-Aldrich) for 1 hour, followed by treatment for 48 hours with MCM and BAY11-7082. The supernatant (containing the potential contributing factors) was stored at −80 °C until an enzyme-linked immunosorbent assay (ELISA) and further experiments were done.

### Macrophage-mediated degenerative response in PBM-irradiated human NP cells that mimic degenerative IVD conditions

**Figure 1:**
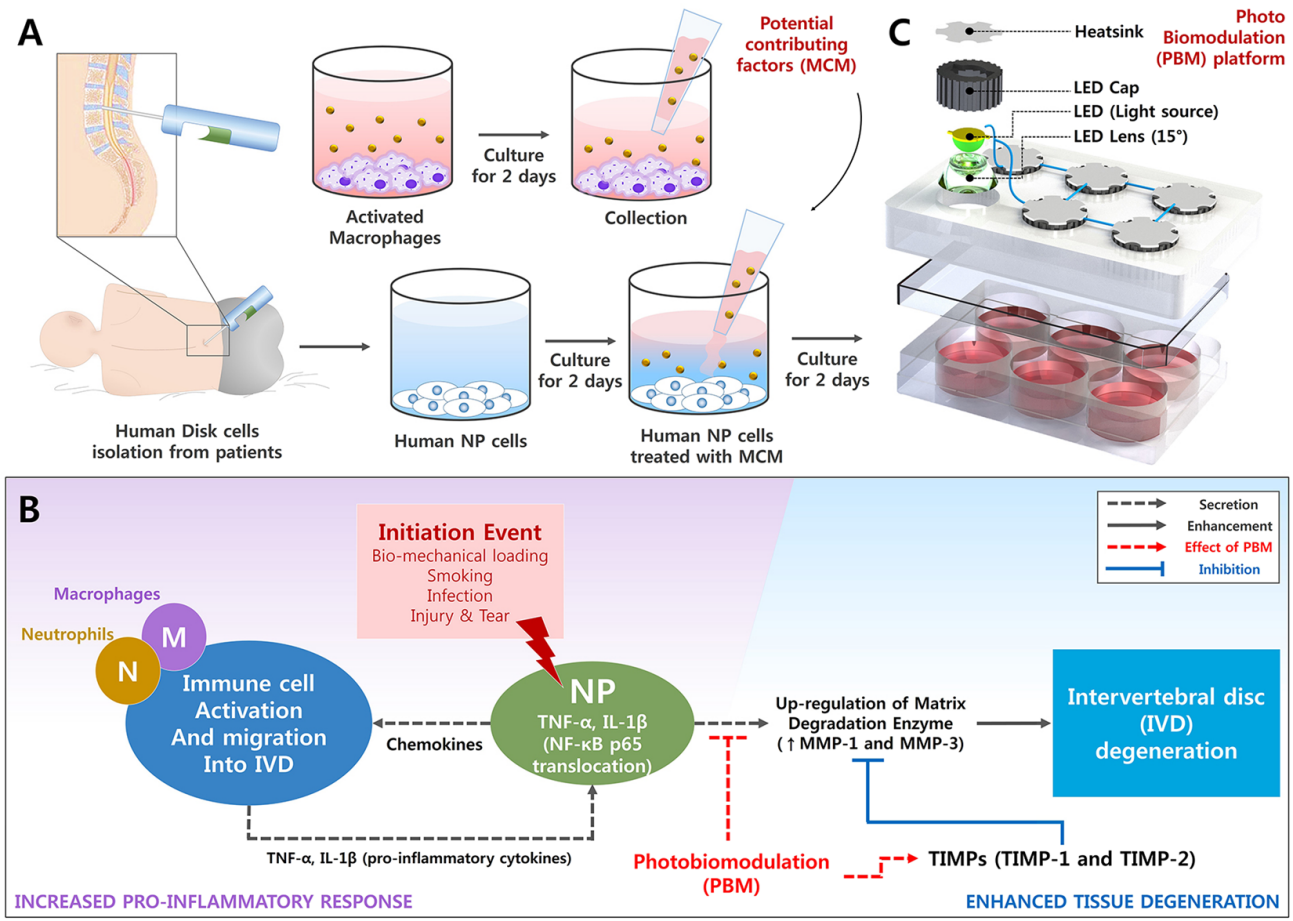
Flow diagram shows application of PBM in human NP cells, possible mechanism of IVD degeneration, and effects of PBM. (**A**) Flow diagram shows degenerative models of human NP cells stimulated by potential contributing factors derived from macrophages. (**B**) Mechanisms of IVD degeneration and therapeutic target sites of PBM (**C**) 3D view of the PBM platform comprising heatsink and LED modules.

Human NP cells were plated at a density of 5 × 10^4^ cells per well in 6-well culture plates containing DMEM/F12 supplemented with 1% FBS and 1% P/S. After 48 hours, the medium was removed and MCM was added for an additional 48 hours. A range of wavelengths (465, 525, and 630 nm) and doses (16, 32, and 64 J/cm2) were used to apply PBM to each separate group. This irradiation parameter was determined by our previous studies^26,27^. The supernatant was then harvested, and the production profiles were analyzed using ELISA. mRNA expression levels were analyzed by qRT-PCR. All the irradiation experiments were performed on a clean surface at 37 °C in a humidified atmosphere with 5% CO2. An indium gallium aluminum phosphide (InGaAIP) light-emitting diode (LED) (630, 525, and 465 nm) (Photron Co., Ltd., Anseong-si, Gyeonggi-do, Korea) was used as light source. We have developed three distinct devices, each for a particular wavelength of LED. The PBM platform was controlled by the ATmega128 microcomputer unit (Mouser Electronics Inc., Kwun Tong, KL, Hong Kong, China) to maintain the atmospheric conditions. Figure 1 depicts the schematic diagram of experimental design for degenerative conditions and the effects of PBM (Fig. 1). The phototherapy and experimental treatment parameters are listed in Tables 1–3.

**Table 1:** PBM parameters.

Parameter	Value
Wavelength [nm]	630 ± 15, 525 ± 5, 465 ± 5
Operating mode	Continuous wave
Luminous flux [lm]	50, 45, 25
Average radiant power [mW]	14.08, 18.00, 25.30
Aperture diameter [cm]	0.6
Beam divergence [deg]	15
Beam profile	Top Hat Beam

**Table 2:** Treatment parameters.

Parameter	Value
Beam spot size at target [cm^2^]	2.78
Irradiance at target [mW/cm^2^]	1.56, 2.00, 2.81
Exposure duration (64 J) [sec]	4542, 3558, 2526
**Distance of LED probe**	
from cell culture plate [cm]	1.8
Area irradiated [cm^2^]	9 (6-well culture plate)
Radiant energy [J/cm^2^]	1.78, 3.56, 7.11

**Table 3:** Experimental group.

Group	Description
(1) Control	Naive human NP cells
(2) Macrophage-conditioned medium (MCM)	Potential contributing factors derived from activated macrophage-like THP-1 cells
(3) Degenerative conditions	Human NP cells exposed to MCM
(4) Degenerative conditions + phototherapy	Human NP cells exposed to MCM with PBM

### Enzyme-linked immunosorbent analysis (ELISA)

The concentrations of IL-1β, TNF-α, MMP-1, MMP-3, TIMP-1, TIMP-2, ADAMTS-4, and ADAMTS-5 were measured in the supernatant using commercially available ELISA kits (R&D Systems) according to the manufacturers’ protocols.

### Quantitative real-time polymerase chain reaction (qPCR)

Human NP cells were lysed with Trizol reagent (Invitrogen), RNA was extracted, and cDNA synthesized (Life Technologies) according to the manufacturer’s instructions. The quantity and quality of the RNA were determined using a Nanodrop 2000 Spectrophotometer (Thermo Scientific). qRT-PCR was performed for *MMP1* and *MMP3* using the SYBR Green PCR Master mix (Applied Biosystems). mRNA expression was analyzed using the 2^−∆∆Ct^ method, in which values were expressed as the mean fold change normalized to that of the housekeeping gene GAPDH. Naive NP cells, and NP cells exposed to MCM, were used as controls for NP cells irradiated by PBM.

### Immunofluorescence staining of nuclear factor kappa-light-chain-enhancer of activated B cell (NF-κB) p65 protein

Human NP cells were plated on a glass-bottom confocal dish and exposed to MCM for 48 h. The cells were fixed with 4% paraformaldehyde, permeabilized with 0.2% Triton X-100 in PBS for 15 min at room temperature, blocked with 5% bovine serum albumin (BSA; Millipore) in PBS, and then incubated with the primary antibodies overnight at 4 °C in 5% BSA. Anti-NF-κB p65 mouse monoclonal antibody (Santa Cruz) was used to detect the NF-κB p65 protein. Goat anti-mouse Alexa 555 secondary antibodies (Invitrogen) and 5% BSA were used for the secondary incubation in PBS for 1 h at room temperature. After washing in PBS, the plate was counterstained with 4′,6-diamidino-2-phenylindol (DAPI, Invitrogen). Images were acquired using the EVOS FL Auto cell imaging system (Thermo Fisher Scientific Inc., USA).

### Total collagen measurement (Sircol assay)

Human NP cells were stimulated with or without MCM for 48 hours. The amount of total soluble collagen in the supernatants was quantified using the Sircol collagen assay (Biocolor, Belfast, UK). Sirius red dye (500 μL), an anionic dye that reacts specifically with the basic side-chains of collagen during assays, was added to 100 μL of the supernatant and incubated with gentle rotation for 30 min at room temperature. The sample and Sirius red dye mixture was centrifuged at 13,475 g for 10 min, and the collagen-dye complex was washed with an acid-salt wash reagent to remove the unbound dye from the surface of the complex. The collagen-dye complex was re-centrifuged at 13,475 g for 10 min, and the precipitate was collected and re-solubilized in 0.5 M sodium hydroxide. The sample was transferred into a 96-microwell plate. The total soluble collagen concentration was estimated using a spectrophotometer at 555 nm (Beckman Coulter, Fullerton, CA, USA).

### Cell cytotoxicity and lactate dehydrogenase assay (LDH)

Measurements of the release of lactate dehydrogenase (LDH) were performed per manufacturer’s instructions. After the cells were exposed to PBM, the exposure medium was collected for quantitating the release of lactate dehydrogenase. Viability was calculated with respect to that of the controls (human NP cells treated with MCM). If the human NP cells were damaged by PBM therapy, these cells would show a tendency toward increased LDH production.

### Statistical analysis

Data were expressed as the mean ± standard deviation for four or five individual experiments. One-way analysis of variance (ANOVA) and Bonferroni’s correction post hoc test were used to assess the differences in the experimental groups. The normal distribution of each subgroup was assessed by the Shapiro-Wilk test. For data not showing normal distribution, we used Kruskal-Wallis with Dunn’s multiple comparison test. All statistical analyses were performed using SPSS software (version 21.3, IBM SPSS Statistics Inc., Chicago, IL, USA). A p-value < 0.05 was considered statistically significant.

## Results

### Activated macrophage-like cells induce degeneration in human NP cells by modulating ECM-modifying enzymes and preferentially distributing the NF-κB p65 protein

To determine whether macrophage-like THP-1 cells secrete proinflammatory cytokines that initiate degeneration of human NP cells, we analyzed the production of IL-1β and TNF-α in MCM using ELISA. Additionally, to determine the effects of NF-κB inhibition on protein and gene expression of ECM-modifying enzymes and total collagen, we treated human NP cells using BAY11-7082, which reduces NF-κB activation by inhibiting the IκBα phosphorylation.

**Figure 2:**
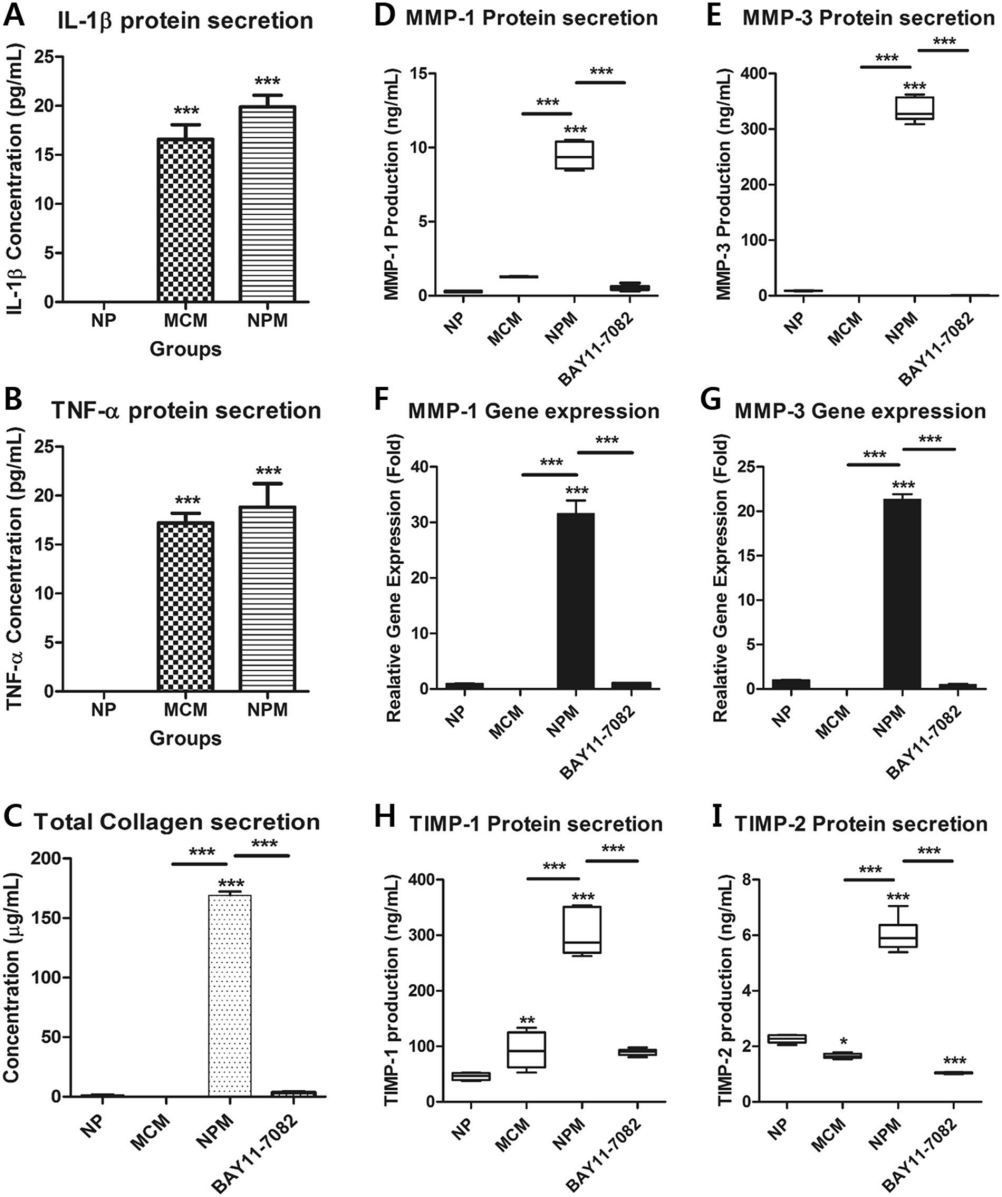
Effects of potential contributing factors, derived from macrophages, on human NP cells with/without BAY11-7082 as an inhibitor of the nuclear factor kappa B (NF-κB) activity. (**A**) Production of IL-1β and (**B**) TNF-α; (**C**) total collagen secretion; (**D**) production of MMP-1 and (**E**) MMP-3. (**F**) Gene expression of *MMP1* and (**G**) *MMP3*. (**H**) Production of TIMP-1 and (**I**) TIMP-2 as endogenous inhibitors of MMP-3 and MMP-1, respectively. Values are mean ± SE of three or four independent experiments. *p < 0.05, **p < 0.01, ***p < 0.001 as compared with NP, and line indicates comparison with each group.

**Figure 3:**
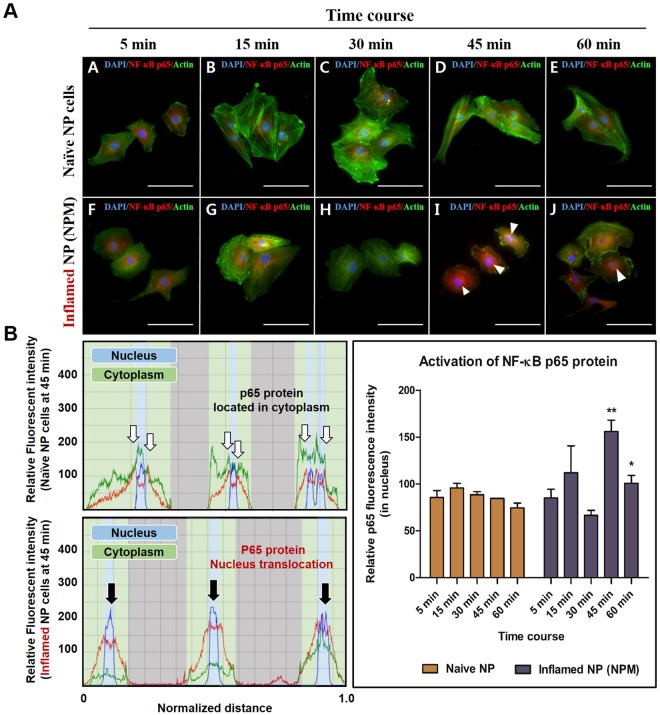
Fluorescence images of preferential expression and translocation of NF-κB p65 protein, occurring in time-dependent manner. (**A**) Fluorescence image of NF-κB p65 protein levels in naïve and inflamed NP cells. (**B**) Quantification of the fluorescence intensity and preferential distribution of NF-κB p50 protein levels in naïve and inflamed NP cells. Human NP cells, exposed to MCM for 45 and 60 min, revealed translocation of p65 protein into the nucleus; this can trigger degenerative conditions because the p65 protein acts as a transcription factor. Scale bar = 100 μm.

MCM showed a significantly higher expression of IL-1β and TNF-α than that in naive NP cells (Fig. 2A,B). To investigate the expression of ECM-modifying enzymes in human NP cells exposed to MCM (NPM), the gene and protein expression of MMP-1, MMP-3, TIMP-1, and TIMP-2 were measured in NPM by qRT-PCR and ELISA. The secretion of collagen, which is upregulated in the early stages of IVD degeneration in human NP cells, was identified by the Sircol assay. The production of MMP-1, MMP-3, TIMP-1, and TIMP-2 in NPM was markedly increased compared with that in naive NP cells (Fig. 2D,E,H,I). NPM also exhibited upregulated genetic expression of *MMP1* and *MMP3* (Fig. 2F,G). Similarly, NPM showed a marked increase in total collagen secretion (Fig. 2C). BAY11-7082 treatment on NPM was able to attenuate the protein production and gene expression of all target factors used in this study compared with NPM (Fig. 2C–I). Additionally, our fluorescence images revealed that NF-κB p65 protein is preferentially distributed in the nucleus under the presence of MCM rather than in the cytoplasm, where it is associated with the catabolic response by acting as a transcription factor, whereas in the absence of MCM, it was present in the cytoplasm (Fig. 3A). Quantitatively, the p65 activity calculated from the average intensity value in inflamed NP cells was shown to have an increasing trend by potential contributing factors derived from macrophages and most of the detected activity was located in the nucleus at 45 and 60 min compared with naïve NP cells (Fig. 3B).

These results indicate that potential contributing factors, derived from activated macrophages, induce degenerative conditions in human NP cells via an increased production of ECM-modifying enzymes, secretion of collagen, and gene expression of catabolic enzymes such as *MMP1* and *MMP3*.

### PBM influences the production and gene expression of MMP-1

To evaluate the effects of PBM on the production and gene expression of MMP-1 and its endogenous inhibitor TIMP-2 in NPM, we treated NPM with PBM in a range of wavelengths (465, 525, and 630 nm) and doses (16, 32, and 64 J/cm^2^).

**Figure 4:**
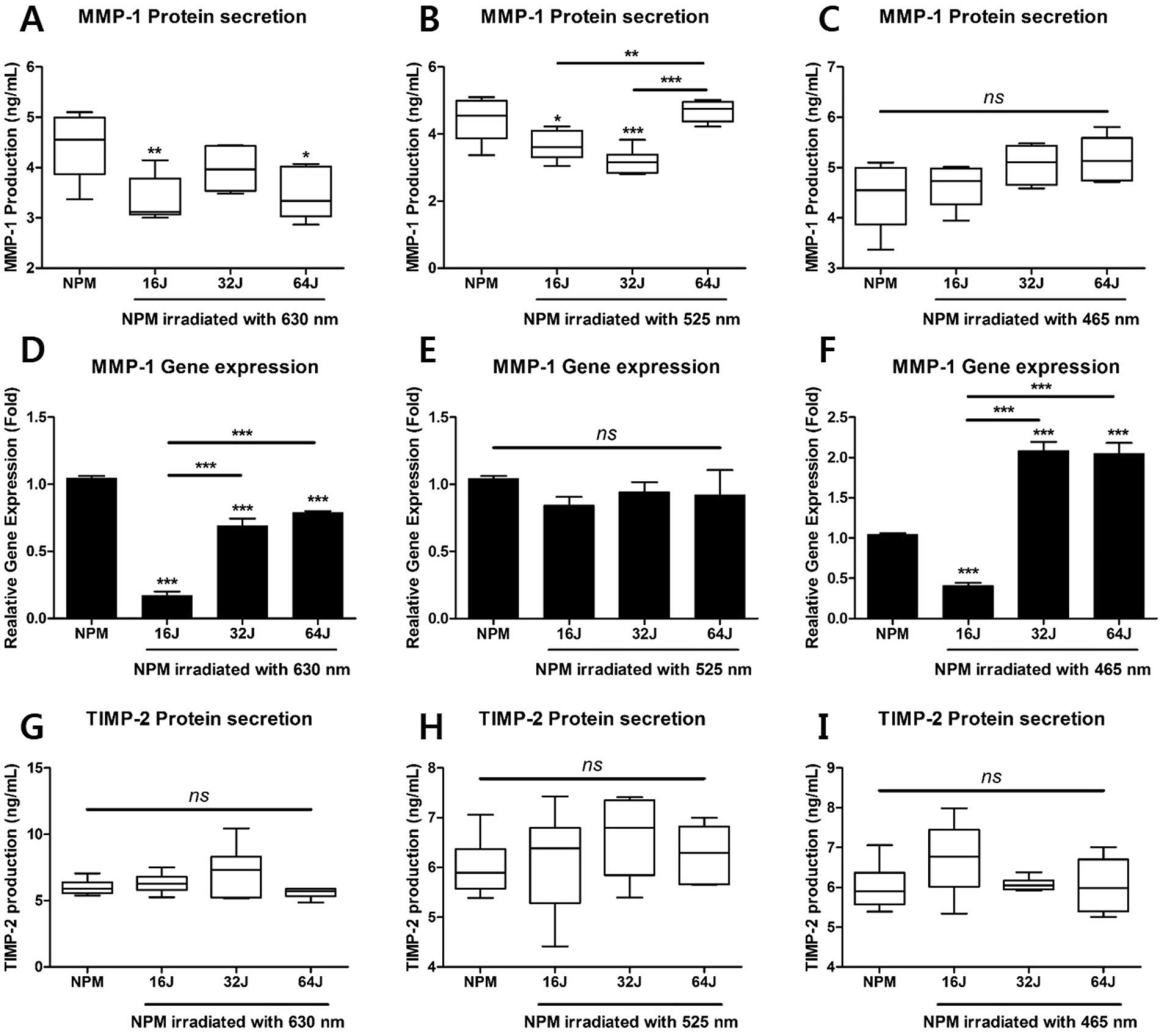
Gene and protein expression of MMP-1, and production of TIMP-2 as endogenous inhibitor of MMP-1, in NPM treated with PBM. (**A**) Production of MMP-1 at 630 nm, (**B**) 525 nm, and (**C**) 465 nm. (**D**) The relative gene expression of MMP1 at 630 nm, (**E**) 525 nm, and (**F**) 465 nm. (**G**) Production of TIMP-2 at 630 nm, (**H**) 525 nm, and (**I**) 465 nm. Values are mean ± SE of three or four independent experiments. *p < 0.05, **p < 0.01, ***p < 0.001 as compared with NP, and line indicates comparison with each group. ns, no significant difference.

First, we measured the gene and protein expression of MMP-1, known as collagenase-1 in IVD tissues, by qRT-PCR and ELISA. Our mRNA results show that all doses of PBM at 630 nm more significantly suppressed the mRNA expression of *MMP1* than that of NPM without PBM (Fig. 4D). These effects result in an inhibited protein production of MMP-1 on NPM, except for that observed at 32 J/cm^2^ (Fig. 4A). PBM at 525 nm with 16 and 32 J/cm^2^ had inhibitory effects in production of MMP-1 (Fig. 4B), but all of doses did not significantly bring about a change in mRNA levels (Fig. 4E). At a wavelength of 465 nm, NP cells were regulated by PBM at the mRNA level at all of the doses (Fig. 4F). However, production of the MMP-1 protein did not change significantly during irradiation with PBM at 465 nm (Fig. 4C). Additionally, there was no difference in the production of TIMP-2 as the endogenous inhibitor of MMP-1 (Fig. 4G–I).

These results demonstrated that PBM at 630 nm with 16 and 64 J/cm^2^ had an inhibitory effect on degenerative NP cells through regulation of both mRNA and protein, and human NP cells were regulated during the production of MMP-1 protein at 525 nm with 16 and 32 J/cm^2^.

### Effects of PBM on gene and protein expression of MMP-3 and its endogenous inhibitor TIMP-1

We used qRT-PCR and ELISA to examine the regulatory effects of PBM on protein production and genetic expression of MMP-3 and its endogenous inhibitor TIMP-1 in NPM.

**Figure 5:**
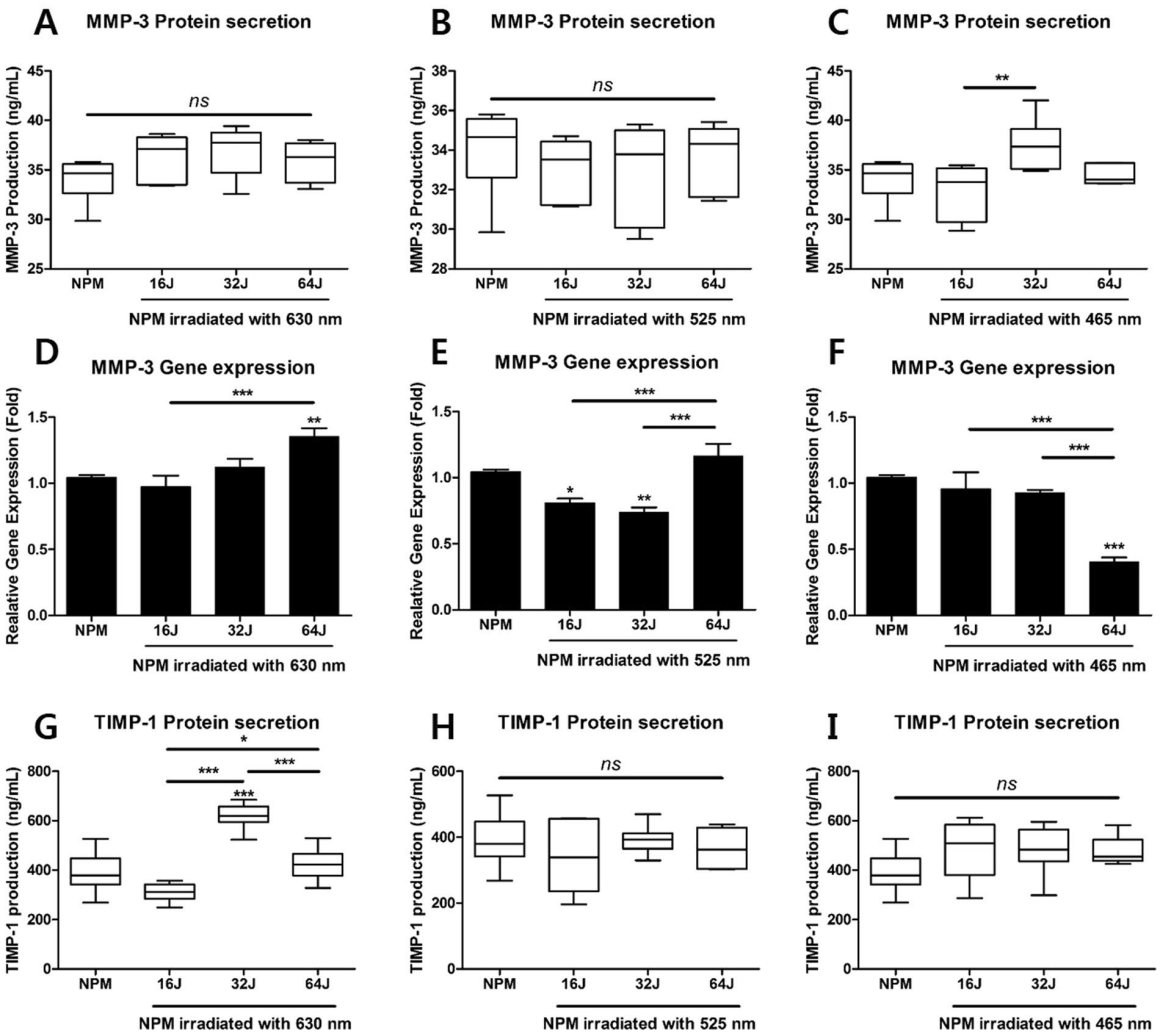
Gene and protein expression of MMP-3 and production of TIMP-1 as endogenous inhibitor of MMP-3, in NPM treated with PBM. (**A**) Production of MMP-3 at 630 nm, (**B**) 525 nm, and (**C**) 465 nm. (**D**) Relative gene expression of MMP3 at 630 nm, (**E**) 525 nm, and (**F**) 465 nm. (**G**) Production of TIMP-1 at 630 nm, (**H**) 525 nm, and (**I**) 465 nm. Values are mean ± SE of three or four independent experiments. *p < 0.05, **p < 0.01, ***p < 0.001, ns, no significant difference, compared with each group.

Our results show that PBM selectively modulated the mRNA expression of *MMP3* at all of the tested wavelengths in dose-dependent manner. However, the change in protein production of MMP-3 was not observed at all the tested wavelengths (Fig. 5A–C). At the wavelengths of 525 and 465 nm, *MMP3* mRNA was significantly down-regulated by PBM at the doses of 16, 32, and 64 J/cm^2^, respectively (Fig. 5E,F); however, the differences in protein production of MMP-3 and TIMP-1 were not significantly different (Fig. 5H,I). Although PBM, at the dose of 64 J/cm^2^ and at 630 nm, induces an upregulation in the mRNA expression of *MMP3* (Fig. 5D), its protein production was not changed. Interestingly, the production of its endogenous inhibitor TIMP-1 was significantly upregulated by PBM at 630 nm with 32 J/cm^2^ (Fig. 5G).

These results indicate that PBM can regulate the genetic expression of *MMP3* in dose- and wavelength-dependent manner, but cannot inhibit the levels of the MMP-3 protein. However, PBM at 32 J/cm^2^ and 630 nm may exert positive effects via elevating the secretion of the TIMP-1 protein; this is associated with decreased MMP-3 activity in degenerative conditions.

### Cytotoxicity assessment using LDH secretion in human NP cells irradiated with PBM

**Figure 6:**
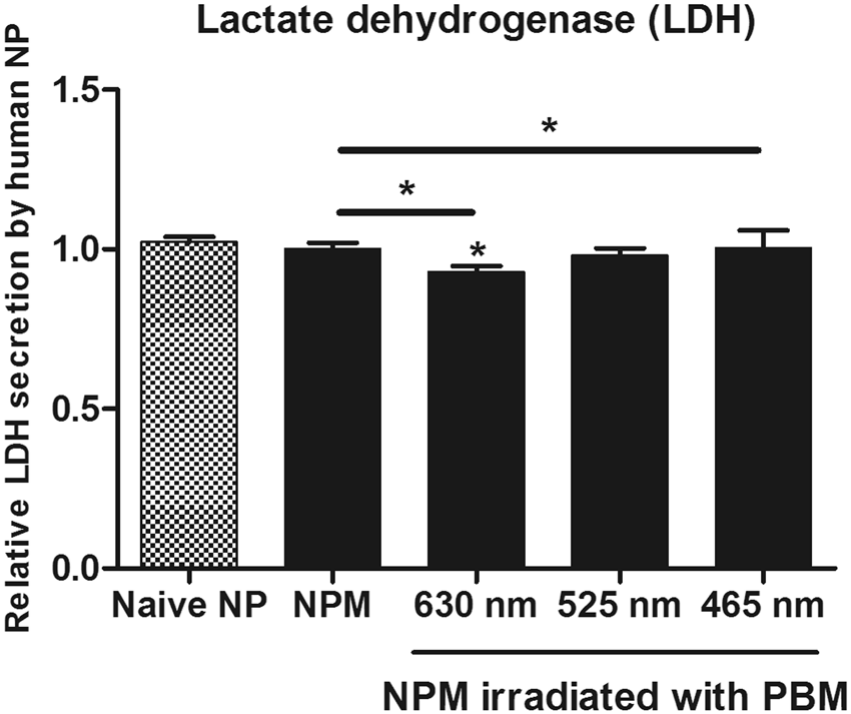
The lactate dehydrogenase (LDH) assay used to assess LDH released by human NP cells treated with PBM. Human NP cells exposed to MCM were irradiated using PBM at 64 J/cm^2^, which is the maximum dose used in this study. The data show that none of the wavelengths, used in this study, were cytotoxic to human NP cells. Values are mean ± SE of three or four independent experiments. *p < 0.05, **p < 0.01, ***p < 0.001 as compared with control, and line indicates comparison with each group.

**Figure 7:**
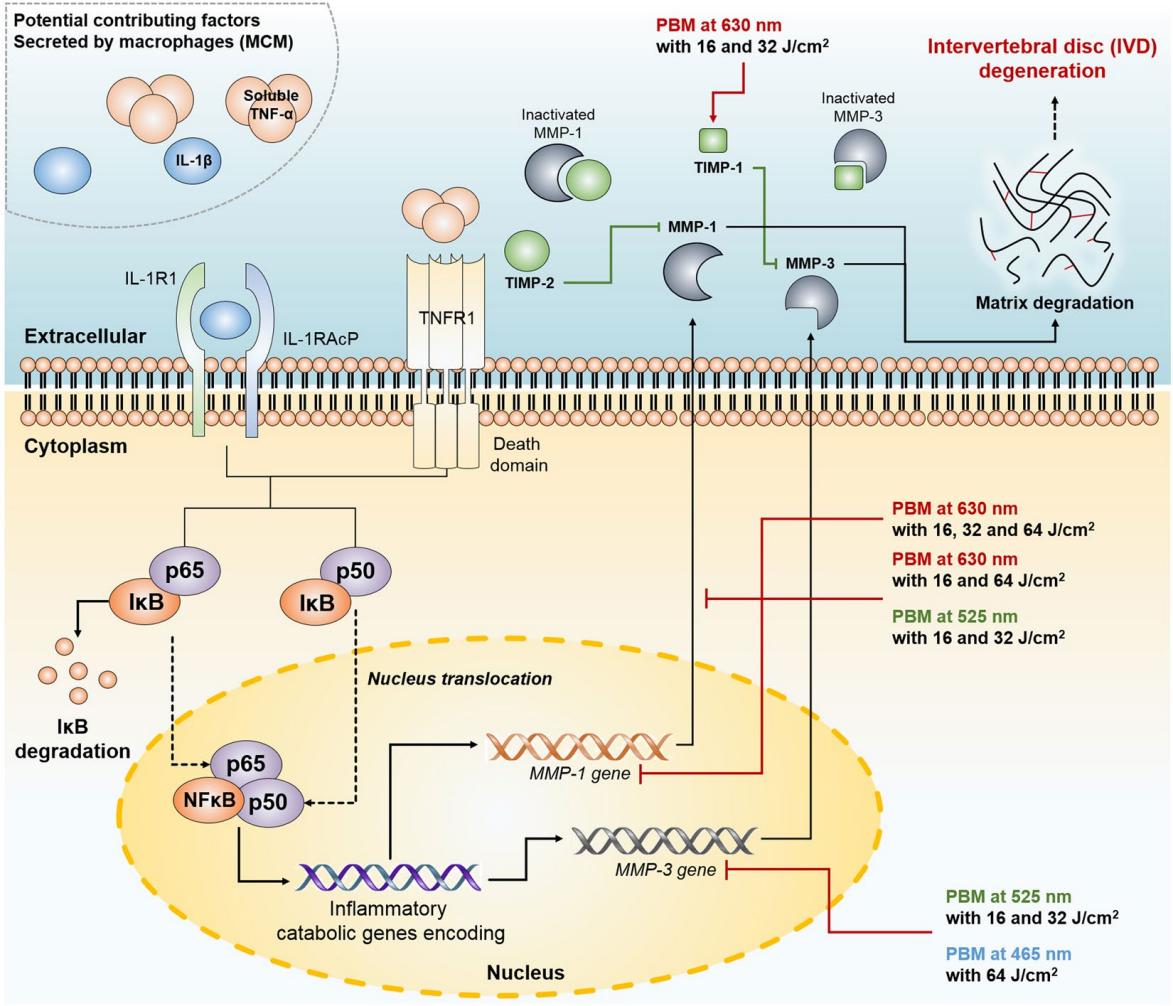
A schematic summary of the IVD degeneration *in vitro* model and effects of PBM on ECM-modifying enzymes in human NP cells. In this IVD degeneration *in vitro* model, macrophage THP-1 like cells express proinflammatory cytokines such as IL-1β and TNF-α. These molecules activate NF-κB downstream signaling, which control expression of inflammatory catabolic genes encoding including *MMP1* and *MMP3* via nucleus translocation of NF-κB (p65 and p50 subunits). Possible effect sites of PBM observed in this study are indicated by red lines. Abbreviations: IκB, inhibitor of nuclear factor κB; IL-1R1, IL-1 receptor 1; IL-1RAcP, IL-1 receptor accessory protein; NFκB, nuclear factor κB; TNF-α, tumor necrosis factor- alpha; IL-1β, interleukin-1beta; TNFR, TNF receptor 1; MMP, matrix metalloproteinase; TIMP, a tissue inhibitor of metalloproteinases.

Measurement of lactate dehydrogenase (LDH) release is a common method used in cytotoxicity assays. Because PBM can damage cells, we tested PBM at the dose of 64 J/cm^2^ at all the assessed wavelengths. In this study, 64 J/cm^2^ was the maximum dose, and it enhanced LDH release from human NP cells exposed to MCM. As shown in Fig. 6, PBM at 525 nm did not significantly up-regulate LDH release from human NP cells. Additionally, irradiation at 630 and 465 nm showed positive effects on the viability of human NP cells (Fig. 6). Together, Fig. 7 depicts a schematic summary of the IVD degeneration *in vitro* model used in this study and effects of PBM on human NP cells (Fig. 7).

## Discussion

We show that potential contributing factors, derived from activated macrophages, induced an upregulation of ECM-modifying enzymes, production of collagen, and preferential activation of the NF-κB p65 protein in human NP cells *in vitro*, mimicking conditions of degenerative IVD. We confirmed that PBM selectively ameliorated these degenerative conditions via modulation of genes and proteins, associated with production of ECM-modifying enzymes, in a dose- and wavelength-dependent manner.

The onset of IVD degeneration is characterized by angiogenesis of vascular structures into otherwise avascular IVD tissues. Using this route, circulating immune cells, including monocytes, neutrophils, and macrophages, infiltrate these IVD tissues. This hypothesis is supported by the results of studies showing infiltration of CD68^+^ macrophages, neutrophils, and T cells (CD4^+^ and CD8^+^), along with invading blood vessels, in herniated discs^16,39^. Proinflammatory cytokines, such as IL-1β and TNF-α, secreted by these infiltrating immune cells, play a major role during progression of the disease. Various studies showed that upregulated expression of proinflammatory cytokines, including IL-1β, TNF-α, IL-6, and IL-17, is observed in degenerative tissues of IVD^16,40–42^. Our results similarly show that potential contributing factors, secreted by activated macrophage THP-1 cells, include proinflammatory cytokines such as IL-1β and TNF-α. IL-1β and TNF-α possess numerous functions, such as stimulating the secretion of inflammatory mediators and inducing the expression of adhesion molecules on endothelial cells; these are responsible for angiogenesis, nerve ingrowth, and chemo-attraction of neutrophils^3^. When these cytokines bind to their receptors including IL-1 receptor type 1 (IL-1R1) and TNF receptor superfamily member 1 A (TNFR1), the resulting intracellular complex leads to activation of the IκB kinase (IKK). IKK phosphorylates the inhibitory IκBα protein, resulting in the nuclear translocation of NF-κB subunits, such as transcription factors p65 and p50, which control the expression of numerous inflammatory and catabolic genes^43–45^. A study reported that inactivation of the NF-κB pathway including inhibition of IKK and stabilization of IκB by prolactin treatment significantly alleviated the progression of IVD degeneration through increasing the collagen components. Our previous studies and other reports demonstrated that stimulation with TNF-α and IL-1β induce the upregulation of various catabolic enzymes, including MMP-1, -2, -3, -9, -13, -14, and a disintegrin and metalloproteinase with thrombospondin motifs (ADMATS)-4 and -5, in IVD cells^18,26,46,47^. These enzymes promote the degradation of ECM components, such as collagen and aggrecan, during IVD degeneration. Clinically, in a healthy state, angiogenesis of vascular structures in the IVD is blocked by high levels of sulfate bonding generated by ECM components and aggrecan^8^. However, in herniated and degenerative IVD, there is a marked increase in the expression of MMPs and ADAMTS. These can induce continuous structural breakdown of ECM components^48,49^. Our results indicate that human NP cells exposed to MCM also showed a dramatic increase in the protein and gene expression of MMP-1 and MMP-3. Furthermore, our immunofluorescence images show that under the influence of potential contributing factors derived from macrophages, the NF-κB p65 protein translocated into the nucleus rather than into the cytoplasm of human NP cells. These results show that potential contributing factors derived from macrophages can induce degenerative conditions in human NP cells via upregulation of ECM-modifying enzymes. Some studies reported that blocking the IL-1β-mediated expression of gene encoding MMPs in IVD cells can restore expression of aggrecan and prevent proteoglycan depletion^50,51^. Hence, regulation of these enzymes may be a biological therapeutic target for the treatment of IVD.

Numerous studies have investigated the effects of PBM in ameliorating or treating various diseases. PBM can lead to reduction of inflammation, cell proliferation, increased synthesis of ATP, and regulation of ECM components^25–27,35,52–54^. Low-level light irradiation suppresses the inflammatory response in human adipose-derived stem cells by modulating the activity of cyclic AMP and NF-κB^55^. Another study showed that treatment with a low-level laser improved tendon healing by regulating MMP activity and collagen synthesis^38,52^. Various studies have shown that PBM alters the gene and protein expression of MMPs and TIMPs. This modulates collagen production via regulation of the intracellular downstream signaling of c-Jun N-terminal kinase-mitogen-activated protein kinase (JNK-MAPK) and extracellular signal-regulated protein kinase (ERK) pathways^53,56,57^.

For these photo-biological effects, a large proportion of photons must be absorbed on electronic absorption band, which is enabled by specific chromophores. Absorption of a particular chromophore depends significantly on the frequency range. The primary chromophore for red light (600–700 nm) is generally cytochrome c oxidase, which is a multi-subunit membrane-associated enzyme that is the end point of mitochondrial respiratory chain^58^. Increasing evidence suggests that flavins, flavor-proteins, or cryptochromes, in case of ROS generation, are photo-acceptors at shorter (visible) wavelengths^59^, such as green and blue light, although the extent of expression of cryptochromes in mammalian cells is yet to be clarified. Additionally, light of this wavelength range is known to be primarily absorbed by opsins (OPNs). The main signaling pathways induced by OPNs are strongly linked to the family of transient receptor potential (TRP) cation channels such as TRPV1 (capsaicin receptor and the vanilloid receptor 1)^60^. TRPV1 has been shown to be activated by various wavelengths of light including green, red, and near-infrared^61,62^. Interestingly, this cation channel is thought to be a major factor in painful IVD. At present, the leading hypothesis to explain symptomatic IVD degeneration is related to the fact that the TRP cation channel in the dorsal root ganglion (DRG) is expressed by neurogenic factors, such as β-nerve growth factor (β-NGF) and brain-derived neurotrophic factor (BDNF) within degenerative IVD tissues^3^.

As discussed above, there is an “optical window” within tissues ensuring effective infiltration of light, and specific photo-acceptor molecules or chromophores for each wavelength may be considered for applying the effects of PBM.

In summary, we hypothesized that PBM may control the gene and protein expression of MMPs or TIMPs in human NP cells. Thus, we investigated the effects of PBM, at various wavelengths (630, 525, and 465 nm) and doses (16, 32, and 64 J/cm^2^), on the regulation of ECM-modifying enzymes in MCM-stimulated human NP cells. Our data show that PBM at 630 and 525 nm successfully and dose-dependently suppressed the expression of MMP-1 without altering TIMP-2 production. Consistent with our results, other study observed that photo irradiation at 660 nm showed upregulation of collagen and down regulation MMP-1 *in vitro*^63^.

PBM at 525 and 465 nm diminished gene expression of *MMP3* in a dose-dependent manner. These results were considered that PBM, applied over a longer period, may alter its protein production. There is possible mechanism for these effects on MMP-3. Recent study showed that phototherapy had the greatest impact on raising mitochondrial membrane potential which will lead to a drop in the levels of reactive oxygen species (ROS) generated in the mitochondria of oxidatively stressed cells^64^. Furthermore, other study showed that inhibition of ROS by N-acetyl-cysteine or diphenylene iodonium significantly suppressed the expression of MMP-3 in lipopolysaccharide (LPS)-stimulated microglia^65^. Thus, we considered that inhibitory effects of PBM on MMP-3 might be modulated by this possible mechanism. However, further studies are needed to elucidate these mechanisms.

PBM at the doses of 32 J/cm^2^ at 630 nm revealed that its inhibitory effects occur via the upregulation of TIMP-1. TIMP-1can attach to alternate or active MMP sites, thereby inhibiting MMPs. Consistent with our result for TIMP-1, recent study showed that phototherapy at 660 nm induced significant increased release of TIMP-1 proteins in stressed fibroblast cells^66^. Later on, an increase in the amount of TIMPs might protect the newly synthesized collagen from proteolytic degradation by MMPs. Our results show that PBM exerts different regulatory effects; these depend not only on the properties of PBM, but also on the target protein. Similar to that, the biphasic dose response or Arndt-Schulz curve in PBM has been shown in various *in vitro* studies and animal models. This phenomenon suggested that insufficient power density that fails to reach the threshold for regulation of gene or protein will have no effect on pathology. In addition, excessive power density may have inhibitory effects or negate the beneficial response induced at optimal power density. Various studies have shown that low- and medium-dose of PBM promoted cell growth, whereas high intensity negated the beneficial effects of PBM in various types of cells^67^. In this study, doses of 16 and 32 J/cm^2^ at 525 nm achieved a significant effect on MMP-1 production and *MMP3* gene expression; this effect was lost when 64 J/cm^2^ was delivered. Additionally, a dose of 16 J/cm^2^ at 465 nm reduced the *MMP1* gene expression levels, whereas higher doses with same frequency promoted it. Doses of 32 J/cm^2^ at 630 and 465 nm were optimal for the modulation of TIMP-1 and MMP-3 production, respectively, although other doses, examined in this study, negated these effects.

Taken together, understanding the mechanisms of additional photo-acceptors and identification of effective doses (considering the biphasic dose-response for target proteins and genes) would be necessary for clinical application. In addition, the parameters used in this study may not be practically applicable in clinics yet. Since light needs to be delivered to the target tissues or cells with sufficient energy, exploring the optimal dose may be required for clinical application. Thus, fusion of PBM irradiation with light delivery system (for example, photosensitizer and/or light guidance system) may be suggested as a strategy for clinical practice.

## Conclusions

In this study, we show that PBM inhibits the macrophage-mediated production of ECM-modifying enzymes in human NP cells in a dose- and wavelength-dependent manner. We conclude that PBM may be a novel tool for the treatment of symptomatic disc degeneration.
